# Supplementary material for: Perinatal Outcomes of Diet Therapy in Gestational Diabetes Mellitus Diagnosed before 24 Gestational Weeks
Source: Nutrients. 2024 May 21;16(11):1553. doi: 10.3390/nu16111553 (PMC11174494; doi:10.3390/nu16111553)
Supplement: Supplementary file 1 [file nutrients-16-01553-s001.zip › Diet_EGDM_STable S1.pdf]

Supplementally Table S1. The nutrition facts of diet therapy for gestational diabetes mellitus at Keio University Hospital.

Calorie intake (kcal)	Protein (g)	Fat (g)	Carbohydrate (g)	Salt (g)	Potassium (mg)	Phosphorus (mg)
1,400	65.1	39.9	195.0	8	2320	1020
1,500	65.1	40.0	219.9	8	2340	1040
1,600	70.0	44.9	229.9	8	2361	1061
1,700	70.1	50.1	240.1	8	2374	1071
1,800	75.1	55.1	255.1	8	2654	1151
1,900	80.1	55.0	270.1	8	2661	1191
2,000	90.1	60.0	270.1	8	2683	1190
2,100	100.0	60.0	300.0	8	3240	1480
2,200	105.0	65.0	305.0	8	3280	1515
