# Supplementary material for: Perinatal Outcomes of Diet Therapy in Gestational Diabetes Mellitus Diagnosed before 24 Gestational Weeks
Source: Nutrients. 2024 May 21;16(11):1553. doi: 10.3390/nu16111553 (PMC11174494; doi:10.3390/nu16111553)
Supplement: Supplementary file 1 [file nutrients-16-01553-s001.zip › Diet_EGDM_STable S2.pdf]

Supplementary Table S2. Characteristics and perinatal outcomes between large for gestational age and non-large for gestational age groups in gestational diabetes mellitus diagnosed before 24 gestational weeks treated with diet therapy.

		LGA (n=35)		Non-LGA (n=274)		p-value
Maternal age at delivery	(years)	36	(31–44)	37	(24–51)	0.86
Nulliparity		23	(66%)	157	(57%)	0.37
Pre-pregnancy BMI	(kg/m^2)	21.8	(18.0–30.1)	21.1	(16.0–36.3)	0.017
Maternal pre-pregnancy BMI category						0.022
Underweight (BMI< 18.5)		2	(6%)	36	(13%)	
Normal weight (18.5≤ BMI< 25.0)		20	(57%)	193	(70%)	
Overweight (25.0≤ BMI< 30.0)		11	(31%)	40	(15%)	
Obese (30≤ BMI)		2	(6%)	5	(2%)	
Family history of diabetes		7	(20%)	56	(20%)	1
Gestational weeks diagnosed GDM	(weeks)	14	(8–23)	14.5	(8–23)	0.41
Random plasma glucose level at the first trimester	(mg/dL)	98	(82–143)	99	(60–169)	0.55
75g OGTT at diagnosed gestational weeks						
Fasting glucose level	(mg/dL)	92	(84–101)	92.5	(67–109)	0.28
1-hour glucose level	(mg/dL)	157	(113–205)	154	(71–242)	0.35
2-hour glucose level	(mg/dL)	134	(93–191)	132	(87–229)	0.92
Initial increase	(mg/dL)	72	(23—115)	59	(-25, 159)	0.52
Subsequent decrease	(mg/dL)	16	(-49, 109)	14	(-75, 104)	0.24
Abnormal values of diagnostic OGTT						
Fasting glucose level (≥ 92 mg/dL)		22	(63%)	168	(61%)	1.00
1-hour glucose level (≥ 180 mg/dL)		8	(23%)	65	(24%)	1.00
2-hour glucose level (≥ 153 mg/dL)		10	(29%)	77	(28%)	1.00
Gestational wight gain expected 40 weeks	(kg)	11	(1.8—17.3)	8.65	(-8.6, 21.3)	0.023
Gestational weeks at delivery	(weeks)	39	(35—40)	38	(23—41)	0.046
Preterm delivery		4	(11%)	35	(13%)	1.00
Cesarean section delivery		16	(46%)	133	(49%)	0.86
Birthweight	(g)	3634	(2834–4526)	2858	(438–3722)	<0.0001
Apgar score 1min		8	(3–9)	8	(1–10)	0.28
Apgar score 5min		9	(6–10)	9	(2–10)	0.28

BMI, body mass index; GDM, gestational diabetes; NGT, normal glucose tolerance.
