# Supplementary material for: Perinatal Outcomes of Diet Therapy in Gestational Diabetes Mellitus Diagnosed before 24 Gestational Weeks
Source: Nutrients. 2024 May 21;16(11):1553. doi: 10.3390/nu16111553 (PMC11174494; doi:10.3390/nu16111553)
Supplement: Supplementary file 1 [file nutrients-16-01553-s001.zip › Diet_EGDM_STable S3.pdf]

Supplementary Table S3. Associations of maternal and perinatal factors with large for gestational age in Diet Early gestational diabetes.

Variable	Unadjusted OR (95%CI)		p-value	Adjusted OR (95%CI)		p-value
Pre-pregnancy BMI (+1 kg/m^2)	1.13	(1.02–1.24)	0.016	1.16	(1.05–1.29)	0.006
Gestational weight gain expected 40 weeks (+1kg)	1.08	(1.00–1.17)	0.048	1.1	(1.02–1.18)	0.016
Gestational weeks at delivery (+1 week)	1.23	(0.98–1.54)	0.076	1.19	(0.98–1.50)	0.085

BMI: body mass index; OR: odds ratio; CI: confidence interval.
