# Supplementary figures and images for: Perinatal Outcomes of Diet Therapy in Gestational Diabetes Mellitus Diagnosed before 24 Gestational Weeks
Source: Nutrients. 2024 May 21;16(11):1553. doi: 10.3390/nu16111553 (PMC11174494; doi:10.3390/nu16111553)

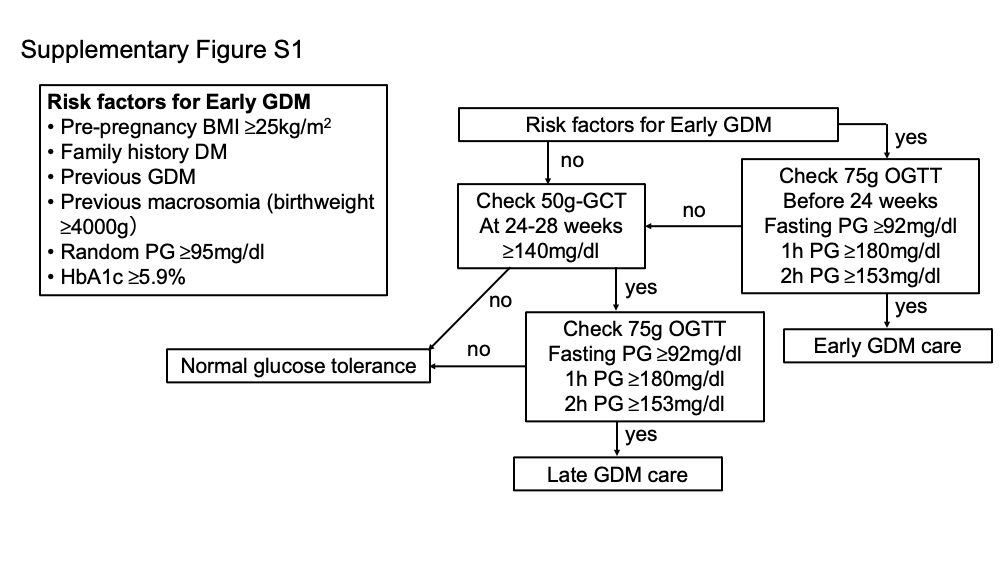

Supplement: Supplementary file 1 [file nutrients-16-01553-s001.zip › Diet_GDM_R1_Sfig S1.tiff]

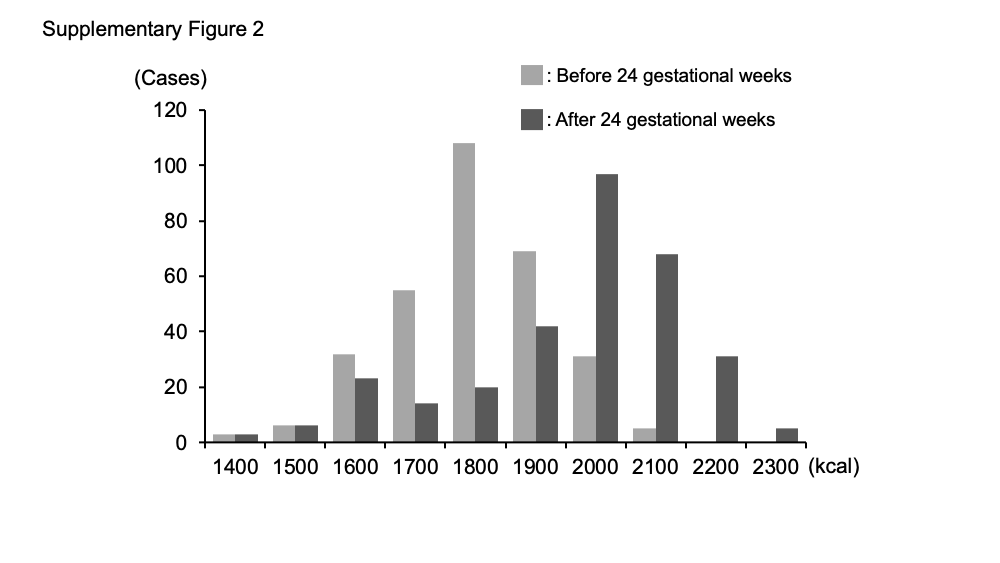

Supplement: Supplementary file 1 [file nutrients-16-01553-s001.zip › Diet_GDM_R1_Sfig S2.tiff]
